# Osteoradionecrosis of the Jaws Due to Teeth Extractions during and after Radiotherapy: A Systematic Review

**DOI:** 10.3390/cancers13225798

**Published:** 2021-11-18

**Authors:** Carlo Lajolo, Cosimo Rupe, Gioele Gioco, Giuseppe Troiano, Romeo Patini, Massimo Petruzzi, Francesco Micciche’, Michele Giuliani

**Affiliations:** 1 Head and Neck Department, Fondazione Policlinico Universitario A. Gemelli IRCCS, 00168 Rome, Italy; carlo.lajolo@unicatt.it (C.L.); cosimorupe@gmail.com (C.R.); 2 School of Dentistry, Università Cattolica del Sacro Cuore, 00168 Rome, Italy; 3 Department of Clinical and Experimental Medicine, University of Foggia, Via Rovelli 50, 71122 Foggia, Italy; giuseppe.troiano@unifg.it (G.T.); michele.giuliani@unifg.it (M.G.); 4 Interdisciplinary Department of Medicine, University of Bari, 70121 Bari, Italy; massimo.petruzzi@uniba.it; 5 Dipartimento di Scienze Radiologiche, Radioterapiche ed Ematologiche, UOC di Radioterapia, Fondazione Policlinico Universitario A. Gemelli IRCCS, 00168 Rome, Italy; francesco.micciche@policlinicogemelli.it; 6 Istituto di Radiologia, Università Cattolica del Sacro Cuore, 00168 Rome, Italy

**Keywords:** osteoradionecrosis, jaw, head and neck cancer, radiotherapy, tooth extraction

## Abstract

**Simple Summary:**

Teeth extractions before or after radiotherapy (RT) could be procedures at high risk for osteoradionecrosis (ORN) onset. This systematic review was performed to investigate the ORN incidence following teeth extractions during and after RT for head and neck (H&N) cancer and to evaluate any other possible risk factor. The results highlight how post-RT teeth extractions are a major risk factor for ORN onset (ORN incidence of 5.8%), especially in the mandible, with a diminishing trend in the last years.

**Abstract:**

Teeth extractions before or after radiotherapy (RT) could be procedures at high risk for osteoradionecrosis (ORN) onset. This systematic review was performed to investigate the ORN incidence following teeth extractions during and after RT for head and neck (H&N) cancer and to evaluate any other possible risk factor. Methods: This systematic review was conducted according to PRISMA protocol, and the PROSPERO registration number was CRD42018079986. An electronic search was performed on the following search engines: PubMed, Scopus, and Web of Science. A cumulative meta-analysis was performed. Results: Two thousand two hundred and eighty-one records were screened, and nine were finally included. This systematic review revealed an ORN incidence of 5.8% (41 patients out of 462, 95% CI = 2.3–9.4); 3 ORN developed in the maxilla. No other clinical risk factors were detected. Conclusion: Post-RT teeth extractions represent a major risk factor for ORN development, especially in the mandible, with a diminishing trend in the last years. Further research on other possible risk factors might improve this evidence.

## 1. Introduction

Among the most common malignancies worldwide, head and neck (H&N) cancers represent the seventh one [1], and almost 75% of patients are treated with radiotherapy (RT), which is either curative or adjuvant or palliative [2]. Unfortunately, RT may cause several side effects, [3] among which osteoradionecrosis (ORN) of the jaws is the most serious.

Signs and symptoms of ORN can vary from pain, sequestration of necrotic bone, and fistulas, to more severe cases with the fracture of the mandible, which can result in sepsis, which is potentially life-threatening, or require major surgical procedures and provoke oral feeding difficulties [4].

ORN can be defined as exposed irradiated bone that fails to heal over a period of three months without evidence of persisting or recurrent tumor; nevertheless, the ORN definition remains a debated topic, due to the following issues: the possibility of ORN onset without bone exposure and the duration of bone exposure necessary to achieve a definite diagnosis, which varies from 1 to 6 months, according to the literature [5,6]. Furthermore, definitions retrieved in literature do not mention the possibility that patients could present jaw bones necrosis due to antiresorptive therapy (medication-related osteonecrosis of the jaws—MRONJ) [7], which may be administered for other tumors and must be excluded in the differential diagnosis or, at least, taken into debt consideration.

Hypovascularity and hypocellularity subsequent to bone irradiation [6] and the following fibro-atrophic process [8] seem to be crucial in the ORN pathogenesis, forming fragile tissues susceptible to necrosis, especially in cases of tissue damage, such as teeth extractions.

Teeth extractions after radiotherapy are recognized as the most important risk factor for the ORN onset [9,10,11,12,13], with a reported incidence ranging between 2% and 22% of patients [14,15], according to the different studied populations and the different diagnostic parameters.

Nabil et coll. (2011) [9] conducted a systematic review that revealed an overall ORN incidence of 7% in patients who underwent tooth extractions after RT; nevertheless, the high number of factors contributing to the ORN pathogenesis (i.e., tumour site, TNM, oncologic therapeutic protocol, oral general status, site of tooth extraction, flap elevation, antibiotics, and hyperbaric oxygen therapy) make the information necessary to prevent ORN onset after tooth extraction insufficient and inadequate, due to the complexity of the topic.

This systematic review was performed to assess (i) the ORN rate following post-radiotherapy tooth extractions; (ii) what is the time-lapse between RT and teeth extraction associated with a lower incidence of ORN; (iii) which other risk factors are associated with the ORN onset; (iv) whether any protocol could prevent or reduce the ORN rate; and (v) whether the ORN rate following the pre-RT tooth extraction is lower than the ORN rate following post-RT tooth extraction.

## 2. Methods

This systematic review was conducted following the Preferred Reporting Items for Systematic Reviews and Meta-Analyses (PRISMA) Statement criteria [16]. PROSPERO Registration was performed, and the following ID was assigned: CRD42018079986.

### 2.1. Inclusion and Exclusion Criteria

Inclusion and exclusion criteria are resumed in Table 1.

### 2.2. Search Strategy and Selection of Studies

An electronic search was performed on the following search engines: PubMed, Scopus, and Web of Science, without specifical filters, from January 1978 to November 2021.

The electronic search strategy was conducted by using a combination of the following MeSH terms and free text words: “Osteoradionecrosis” AND “Dentistry”, “Osteoradionecrosis” AND “Prevention”, “Osteoradionecrosis” AND “Tooth Extraction”, and “Osteoradionecrosis” AND “Tooth Removal”.

**Figure 1:**
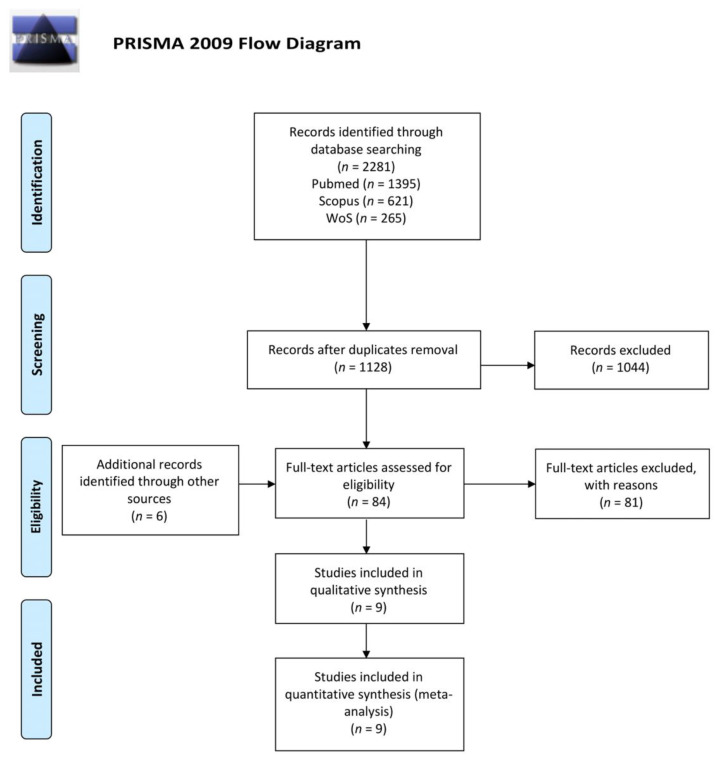
PRISMA flow-diagram of the selection process. Nine articles were finally included in the systematic review and meta-analysis. Adapted from Moher, D et al. (2010) [16]. For more information, visit www.prisma-statement.org (Accessed on 15 November 2021).

Two reviewers (G.T. and G.G.) assessed the studies’ eligibility in a standardized independent manner. If there was any disagreement, it was evaluated by a third reviewer (C.L.) for the final decision. The screening process was conducted according to the PRISMA flow-diagram (Figure 1). A manual search was also conducted on the following journals: Oral Oncology, Clinical Oral Investigations, Oral Diseases, and European Journal of Oral Sciences. In addition, reference lists of the included articles were manually searched, in order to retrieve any possible full-length papers which could be included.

### 2.3. Data Collection

General information on the included papers (i.e., study design, year of publication, country, number of patients, ORN definition, and diagnostic process) and data related to patients (i.e., age and gender, tooth extraction protocol, extraction-related ORN, and other possible risk factors) were collected into a customized table.

### 2.4. Risk of Bias Assessment

The risk of bias assessment was performed throughout the modified Newcastle Ottawa scale [17] and the Jadad scale [18] (File S1, Supplementary Materials) by 2 reviewers (C.R. and C.L.). In case of disagreement, the final assessment was performed by a third reviewer (G.T.).

### 2.5. Statistical Analysis

A cumulative meta-analysis was performed with a random effects model in accordance to DerSimonian–Laird method. The pooled proportion (PP) of the rate of ORN occurrence was calculated. The results of the meta-analysis were presented throughout a forest plot graph. The software Open Meta-Analyst version 10 was used to perform the statistical analysis.

## 3. Results

### 3.1. Results of Search and Study Selection

The electronic search provided 2281 records (PubMed: 1395 papers, Scopus: 621 papers, Web of Science: 265 papers), and 84 papers were selected for full-paper evaluation. The manual search retrieved six additional articles which underwent a full-text evaluation; providing a total of 90 reviewed papers. Nine articles fulfilled the inclusion criteria and, thus, were included in qualitative and quantitative synthesis [11,12,14,15,19,20,21,22,23]. Table S1 (Supplementary Materials) reports the reasons for the exclusion of the other 81 full-length papers.The selection process is reported as a flow-diagram, following the PRISMA guidelines, in Figure 1.

### 3.2. Study Characteristics and Summary of Results

This systematic review includes seven retrospective cohort studies, one prospective study, and one clinical trial.

General information on the included papers (i.e., study design, year of publication, country, number of patients, ORN definition, and diagnostic process) is reported in Table 2.

**Table 2:** Population data of the selected articles: a total of 462 subjects underwent teeth extractions after RT.

Study	Study Design	Included Patients			Mean Age	Mean Follow-Up	RT Technique			Mean Dose ^§^	Patients Receiving Tooth Extraction	Cases of ORN	ORN Due to Tooth Extraction
		Tot	M	F	Years	Months	EBR	IMRT	BT	Gy	n.	n.	n.
Morrish et al., 1981 [19]	R	100	60	40	65	23	100	0	0	66	18	22	9 ^a^
Beumer et al., 1983 [15]	R	72	-	-	-	*	72	0	0	-	72	16	16 ^a^
Marx et al., 1985 [20]	RCT	74	-	-	-	*	-	-	-	68	74	13	13 ^b^
Epstein et al., 1987 [21]	R	146	103	43	54.7	60	140	0	6	-	54	8	3 ^a^
Maxymiw et al., 1991 [12]	P	72	-	-	57.4	57.6	72	0	0	50	72	0	0 ^b^
Lambert et al., 1997 [22]	R	47	-	-	-	35.3	-	-	-	60.6	46	0	0 ^b^
David et al., 2001 [23]	R	24	13	11	61	10.3	-	-	-	-	24	0	0 ^b^
Ben-David et al., 2007 [14]	R	176	128	48	55	35	0	176	0	54.6	13	0	0 ^c^
Al-Bazie et al., 2016 [11]	R	89	55	34	41.8	63	-	-	-	65.4	89	0	0 ^b^

* Although it was not possible to identify a mean value, the study was included because every patient received a follow-up of at least six months. ^§^ The prescribed dose to the tissues affected by the neoplasm. ^a^ Bone exposure longer than 3 months. ^b^ Bone exposure longer than 6 months. ^c^ Bone exposure is present in 2 consecutive follow-ups (6–8 weeks for the first two years, 3–4 months after the first 2 years). Abbreviations: Tot, Total; M, Male; F, Female; n., number; RCT, Randomized Clinical Trial; P, prospective; R, Retrospective; RT, radiotherapy; ORN, osteoradionecrosis; EBR, External Beam Radiotherapy; IMRT, Intensity Modulated Radiation Therapy; BT, Brachytherapy; Gy, Gray.

Specific information regarding patients who underwent teeth extractions is presented in Table 3.

**Table 3:** Characteristics of patients who underwent teeth extraction: among 462 patients who received tooth extractions after RT, 41 ORN were diagnosed.

Study	Patients	Time from RT to Teeth Extraction	N. of Teeth Extraction	ORN Patients	ORN Sites		
		Months			Tot	Maxilla	Mandible
Morrish et al., 1981 [19]	18	-	-	9	9	-	-
Beumer et al., 1983 [15]	72	31	27	16	16	3	13
Marx et al., 1985 [20]	74	-	291	13	35	0	35
Epstein et al., 1987 [21]	54	32.4	173	3	3	0	3
Maxymiw et al., 1991 [12]	72	-	449	0	0	0	0
Lambert et al., 1997 [22]	46	-	704	0	0	0	0
David et al., 2001 [23]	24	-	54	0	0	0	0
Ben-David et al., 2007 [14]	13	-	-	0	0	0	0
Al-Bazie et al., 2016 [11]	89	15	232	0	0	0	0

Abbreviations: n., number; Tot, Total; ORN, osteoradionecrosis; RT, radiotherapy.

**Figure 2:**
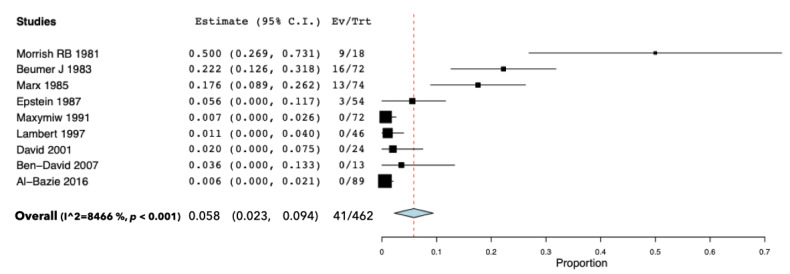
One-way forest plot of the selected articles shows the PP of the incidence of ORN in irradiated patients receiving teeth extractions during and after RT. Abbreviations: CI, Confidence Interval; Ev, Events; Trt, Total of Patients receiving teeth extractions; I^2^, Higgins’ Hindex.

Teeth extractions were performed during and after RT on 462 patients out of a total of 800 subjects suffering from H&N cancer. Overall, among these patients, 41 received an ORN diagnosis at the extraction site in a mean follow-up of 40.6 months. The meta-analysis revealed a 5.8% ORN incidence (95% CI = 2.3–9.4, *p* < 0.001). The analysis showed the presence of a high rate of heterogeneity between the studies (I^2^ = 8466%). The pooled proportion (PP) and the box plot of the included articles are reported in Figure 2.

Three patients out of 41 developed ORN in the maxilla, while all of the others affected the mandible. Table S2 (Supplementary Materials) shows the details of reported ORN, although only few data could be retrieved.

### 3.3. Risk of Bias Assessment

The risk of bias assessment for the included papers is reported in Table 4. The methodological quality of the included studies was dis-homogeneous. Four articles out of nine reached a high score, such as Al-Bazie et al. (2016) [11,14], whereas others had an elevated risk of bias. Furthermore, the selection risk of bias was low, since all the inclusion criteria were strict, including only studies performed on a population of irradiated H&N cancer patients who received teeth extractions during and after RT. The shortcomings mostly concerned the comparability and the outcomes domains: in fact, no studies reported other confounders (i.e., antiresorptive drugs), and only a few studies reached one year of follow-up after teeth extractions and outlined the drop-out rate.

**Table 4:** Modified Newcastle-Ottawa Score and Jadad scale.

**Cohort Studies**	**Selection**				**Comparability**			**Outcome**			**Modified Newcastle-Ottawa Score (Risk of Bias)**
Author	Representativeness of cohort	Selection of non-exposed cohort	Ascertainment of exposure	Outcome of interest not present at onset	Control of confounding factors (extraction)	Control of confounding factors (field of radiation, timing, extraction protocol)		Assessment of outcome	Length of follow-up	Lost to follow-up	
Morrish et al., 1981 [19]	x	x	x	x	x			x	x	x	8
Beumer et al., 1983 [15]	x		x	x		x		x	x	x	7
Epstein et al., 1987 [21]	x	x	x	x	x	x		x			7
Maxymiw et al., 1991 [12]	x		x	x	x			x	x	x	7
Lambert et al., 1997 [22]	x		x	x	x			x	x		6
David et al., 2001 [23]	x		x	x	x	x		x	x	x	8
Ben-David et al., 2007 [14]	x	x	x	x	x			x	x	x	8
Al-Bazie et al., 2016 [11]	x		x	x	x	x		x	x	x	8
**RCT Studies**	**Randomization**				**Blinding**			**Description of Withdrawal and Dropouts**			**Jadad Scale**
Author	1 point if randomization is mentioned	1 point if the method of randomization is appropriate		Deduct 1 point if the method of randomization is inappropriate	1 point if blinding is mentioned	1 point if the method of blinding is appropriate	Deduct 1 point if the method of blinding is inappropriate	1 point if withdrawal and dropouts are described			
Marx et al., 1985 [20]	x										

### 3.4. Results of Individual Studies

Results of individual studies among patients who underwent teeth extraction after radiotherapy are reported in Table 2 and Table 3.

### 3.5. Excluded Studies

The reasons for the exclusion of the other 81 full-length papers are summarized in the Table S1 (Supplementary Materials), available electronically.

In particular, 22 studies did not reach an adequate sample size to be included; 17 studies provided an inadequate definition or diagnosis of ORN; 11 studies had a design not fulfilling the inclusion criteria (reviews, letters to editor); 15 studies analyzed a cohort not representative of the whole population of patients undergoing tooth extractions during or after RT; seven studies did not reach an adequate follow-up (six months after tooth extraction); nine studies diagnosed ORN cases, but it was not clear whether the ORN developed at post-extraction sites.

The study conducted by Schweiger et al. (1987) [24] was remarkable; nevertheless, it did not fulfill the inclusion criteria: the authors made an ORN diagnosis after one month of bone exposure. Notably, a medical examination conducted one month after tooth extraction may overestimate the ORN rate. In fact, the authors reported a higher risk of ORN incidence (8%) following post-RT dental extractions.

The study conducted by Saito et al. (2021) [25] was well conducted; nevertheless, as the authors declared in their discussion section, it was not possible to distinguish if ORN was present at the moment of the extraction or if it was a consequence of the post-RT dental extraction. This could have led to an overrating of ORN incidence (28.1%, as reported by the authors).

Another recent study, performed by Kubota et al., 2021 [26], showed good methodology. Nevertheless, the authors did not specify whether the ORN developed at the post-RT extraction site.

## 4. Discussion

The role of dentists in the H&N cancer supportive therapy is becoming fundamental. The main objectives of dental treatment in these patients, before radiotherapy, are the removal of oral foci and, after radiotherapy, the prevention and therapy of dental diseases and the side-effects of radio-chemotherapy involving the oral cavity. Development of more accurate radiotherapy techniques (e.g., IMRT) has decreased the number of side-effects in the oro-maxillofacial district [27]; nevertheless, ORN remains the most important event, and together with severe mucositis, which sometimes undermines a patient’s life, it can occur in 2% to 22% of irradiated subjects [14,15]. Since teeth extractions performed after the RT represent the main risk factor for ORN onset, dentists should prevent dental diseases to minimize the number of extractions after the RT, and in the case where extraction is necessary, dentists should apply specific protocols to decrease the risk of the onset of ORN.

However, the possible progression of dental diseases, precipitated by the consequences of RT on oral and maxillofacial tissues (e.g., radio-induced caries), and the increase in life expectancy determine the possibility to perform dental extractions in patients who received radiotherapy for H&N cancer [1,28,29]. This systematic review showed an ORN rate of 5.8% in patients undergoing tooth extractions after RT, in accordance with the systematic review conducted by Nabil et coll. (2011) [9]. Comparing the final data obtained from this systematic review (5.8% of ORN in post-RT) with those of extractions performed before radiotherapy (2.2%), reported in a systematic review already conducted by our research group [30], it seems reasonable to consider post-RT extractions as a high-risk procedure and suggest performing them before starting RT. These results are in contrast with the findings emerging from another systematic review, which did not retrieve statistically significant differences in the ORN risk between patients undergoing tooth extractions before RT and patients undergoing tooth extractions after RT [31]. Although it is not easy to find an explanation for these differences in the results, it could be related to less restrictive inclusion and exclusion criteria adopted by Beaumont S et al. (2021). Nevertheless, both the reviews show how a thorough analysis of the risk factors needs to be performed, by means of new clinical trials, in order to reach a better understanding of the pathogenesis of ORN, as further discussed in the discussion section.

However, if we analyze the incidence of ORN in the papers included in this review, it is very uneven: notably, as presented in Figure 2, incidence varies from 50% to 5.6% in the articles prior to 1990 and is up to 0% in articles published from 1990 to today. Therefore, it seems that post-RT extractions no longer involve this risk, unlike pre-RT extractions, which despite a decreasing trend, still show a certain percentage of ORN, and this has been observed in recent studies too (e.g., 7.6% in Schuurhuis, 2011 and 13% in Batstone, 2012) [32,33]. Nevertheless, a recent study conducted by Kubota H et al. (2021) reported an ORN rate of 7.5% in patients who underwent radiotherapy during the last decade [26]. Further studies are needed in order to better clarify the real incidence of ORN. This different frequency of ORN for post-RT extractions, between studies conducted before and after 1990, appears notable but is difficult to fully understand.

Possible explanations are the introduction of the more advanced technique (IMRT) that could have contributed to the progressive reduction of this incidence. IMRT selectively irradiates the tumor, giving a significantly lower dose to healthy tissues. In the 1980s, the transition from traditional 2D to conformed 3D (3DCRT) treatment represented a critical advance in RT. In 3DCRT, simulation and treatment planning are based on computerized tomography (CT), reaching a precise definition of the area affected by neoplastic disease and a more accurate dose calculation. Afterwards, the introduction of IMRT, a highly specialized typology of conformative therapy, through the modulation of the beam flow, allowed the irradiation of the target site with a non-uniform intensity, increasing the dose only to cancer tissues. Furthermore, it allowed the use of multiple irradiation planes, including oblique and not coplanar planes, which together with the use of multilamellar collimators, ensure adequate irradiation of tumor tissues and the saving of healthy tissues, including alveolar bone. However, in many of the studies analyzed, the radiotherapy technique used was unknown.

Furthermore, the increased involvement of dentists in the management of H&N cancer patients could have improved oral conditions of patients post-RT: careful dental treatment before the beginning of RT (e.g., extraction of all teeth with uncertain prognosis), a thorough dental follow-up after the RT (e.g., interception of any possible dental diseases at early stages), and supportive therapies (i.e., oral hygiene recalls and professional fluoride therapy) may contribute to a better oral health after RT. The result of such careful management could mean (1) a lower number of extractions per patient, (2) less inflamed/infected foci, (3) a more accurate extraction planning, and (4) better general oral health conditions.

Our first consideration focuses on the critical issues of the definition and diagnosis of ORN. In accordance with the literature published in the last 15 years, we included only those studies that provided a clear definition of ORN and in which the ORN was diagnosed in the case of irradiated bone, exposed in the oral cavity, for a minimum of three months, with no local recurrences [5,6,34]. Most of the excluded studies, analyzed in full-text, provided no clear definition of the disease. We considered it essential that a clear definition of ORN was present in the study; in the literature, there are several definitions which differ from each other in the length of time of bone exposure and about the bone exposure as a main sign of ORN diagnosis. Although the bone exposure has to linger in post-extractive alveoli for a period of time such as to exclude delayed healing of the alveolus (i.e., dry socket), there is no agreement in defining the post-extraction time interval after which an ORN may be diagnosed. A short time interval could notably overestimate the real ORN rate; by contrast, a long time interval could underestimate the real rate of ORN because some ORN can heal spontaneously, going through bone sequestration, and therefore not be correctly diagnosed. Furthermore, some authors described the possibility that ORN occurs even without bone exposure [35]. Therefore, considering exposed bone as the only sign of ORN, the ORN rate could be underestimated due to a misdiagnosis or to a diagnostic delay. Further research should provide a clear definition of ORN so that it would be possible to compare the results and provide data with a stronger level of evidence.

Another relevant methodological bias that we found from the analysis of the literature concerns the outcome: most of the studies provided information on the number of patients with ORN without providing any information on the number of sites affected by ORN. Considering that ORN may occur in more than one site in the same patient, further research might provide a precise indication of the sites affected by ORN in relation to the post-extraction site. Moreover, to provide a specific risk of ORN onset at post-extractive alveoli, the studies should provide more precise information on the affected sites subjected to extraction (in the irradiated patient population). Contrariwise, most of the studies provided no information on either the sites undergoing post-RT extractions or on the number of post-extractive sites affected by ORN, except Marx et al. (1995) [20].

Some noteworthy clinical considerations concern the anatomical site of tooth extraction. Mandibular jaw appears to be a risk factor of ORN onset following teeth extractions. This systematic review reported only three cases of ORN in the maxilla, while all the other cases developed in the mandible. Unfortunately, it was not possible to clarify the ORN risk related to anatomical site, since the included articles did not report data regarding the anatomical site of extracted teeth in the overall population undergoing RT. Another clinical consideration concerns the surgical technique adopted for the extraction of teeth in patients that received irradiation. Non-surgical extractions are less invasive; however, the lifting of a flap allows the closure of the post-extraction site by first intention and the possibility to modify the bone morphology when necessary. Nowadays, little is known regarding whether any innovative surgical technique can decrease the ORN risk. Marx et coll. (1985) and Maxymiw et coll. (1991) performed all teeth extractions without lifting a flap [12,20]. The ORN rate found by these authors was somewhat discordant: Marx diagnosed 35 ORN out of 291 extracted teeth, and Maxymiw diagnosed no ORN out of 449 extracted teeth. However, the other included articles did not report sufficient data regarding teeth extraction techniques. Further studies are necessary to confirm whether the extraction technique influences the risk of ORN.

Another little-known aspect concerns the reasons to perform dental extractions in this specific cohort of patients: none of the included articles provided information on this matter. Notably, an assessment should be performed as to whether the motivation for a tooth to be extracted could favor the onset of ORN, bearing in mind that the non-extraction of teeth affected by inflammatory-infectious processes could represent a trigger for the onset of ORN, similar to what occurs for MRONJ [36]. By contrast, it seems reasonable that extractions of teeth affected by an inflammatory-infectious process may represent a higher ORN risk procedure. However, post-irradiated alveolar bone could be affected by spontaneous ORN, miming in the early stages an inflammatory-infectious process, overestimating the risk of ORN consequent to post-RT extraction. The articles included in this review do not provide information regarding this topic.

A necessary consideration is relative to the dose received by the post-extraction sites, which could be considered a risk factor for ORN onset. The patients affected by ORN received an average dose of 68 Gy. Unfortunately, it was not possible to define a threshold, since the included articles did not provide information for the specific post-extraction alveoli. Nevertheless, a reasonable opinion is that high-dose radiation therapy increases the risk of ORN.

A highly debated topic in the literature concerns the identification of a time interval after the end of the RT, beyond which the surgical procedures may be safer or associated with a lower risk. Although a reasonable judgement seems to be that postponing the extraction can reduce the risk of ORN, alterations of bone metabolism could persist or worsen several years after the end of radiation therapy. This systematic review showed that patients who developed ORN had a mean time interval from RT to dental extractions longer than the whole population (33 months vs. 24.7 months); these data, contrariwise to general opinion, seem to suggest that a longer time-lapse between RT and ORN could not prevent the ORN onset. However, this information was reported in only two of the four studies that diagnosed ORNs and refers to average values. Specifically, Beumer et al. (1983) [15] conducted dental extractions at different time intervals (7–60 months), and the time-interval from RT to dental extraction was not associated with a higher ORN risk. Although the most recent evidence seems to confirm this result [31] and some authors suggest performing tooth extractions in the immediate post-RT period [26], it is important to consider the possible existence of a “bimodal pattern” of RT damage, showing two different peaks of risk: 12 months after the end of RT and 24–60 months after RT [10]. At present, no controlled studies allow a conclusion regarding the existence of a time interval that reduces the risk of ORN; therefore, this topic warrants further investigation.

Among the risk factors to be evaluated in the estimate of the onset of ORN, the influence of any previous or ongoing medical therapy that may enhance the risk must also be considered. The increased number of patients undergoing medical treatments with antiresorptive, antiangiogenetic, and biological drugs (e.g., denosumab, bisphosphonates) for oncological or metabolic reasons makes it necessary to conduct an accurate interview of the medical history of each patient [37]. Studies included in this review provided no information on this regard. A critical clinical consideration pertains to the different peri-operative medical support protocols reported in the literature to reduce the ORN risk. Among those protocols, antibiotics associated with antiseptic rinses are the most used. There is strong evidence that the deeper zones of necrotic bone are colonized by bacteria of the oral district, so much so that the pathogenetic idea of aseptic necrosis has been repeatedly challenged over time. In the study conducted by Al-Bazie et al. (2016) [11] and Maxymiw et al. (1991) [12], the antibiotic prophylaxis with amoxicillin and penicillin V was included in the protocol and was effective in the prevention of ORN, reporting an ORN rate of 0% (0 ORN out of 161 patients). Additionally, Marx et al. (1985) [20] and Epstein et al. (1987) [21] performed antibiotic prophylaxis; however, their studies showed a higher ORN rate of 35.4% and 5.56%, respectively (altogether, 16 ORN cases out of 91 patients, indicating an ORN rate of 17.58%). Further studies with a larger sample size are therefore needed to clarify the real usefulness of antibiotics in preventing ORN.

Hyperbaric oxygen therapy (HBO) is another peri-operative support provided. The rationale for using HBO is based on the impact of an increased amount of oxygen on hypoxic tissues. Locally, HBO increases the amount of growth factors, including those playing an active role in angiogenesis. Oxygen can also promote an antibacterial effect on the trauma site. Based on the available evidence, the effectiveness of HBO in preventing ORN is debated [38,39]. The articles included in this systematic review did not provide sufficient data regarding the effectiveness of HBO. Further trials are needed to resolve the controversy [37].

Another consideration should be done among new drugs proposed for ORN medical therapy (i.e., pentoxifylline, tocopherol) that could also represent a new approach to the prevention of ORNs [40]. Thus far, none of the studies has analyzed this aspect: future clinical studies might evaluate the preventive role of these drugs for ORN onset.

## 5. Conclusions

This systematic review highlights that dental extractions after RT are procedures at high risk of ORN, especially in the mandible. It was impossible to draw definitive conclusions about other clinical risk factors, including the time-lapse to respect between RT and tooth extractions. Data gathered from the analyzed literature presented a higher rate of ORN (5.8%) when compared with extractions performed before RT (2.2%) [30]; even if the general trend of ORN is decreasing for both pre- and post-RT extractions, studies performed on extraction after RT presented a peculiar bimodal trend: studies before 1990 show a much higher ORN rate compared with those performed after 1990, which are proximate to 0%. Reasons for this bimodal behaviour are not completely understood; possible explanations are that the introduction of the more advanced radiotherapy techniques and the greatest role of the dental clinician for H&N cancer supportive therapy could have improved oral conditions of patients after RT. Further research among other possible risk factors should be conducted to investigate their role in ORN development.

## Figures and Tables

**Table 1:** Inclusion and exclusion criteria adopted for this systematic review.

**Inclusion Criteria**
Full papers, literature in English language, published after 1978 in peer-reviewed journals
Observational clinical studies, both prospective and retrospective (cohort and case-control), and RCTs
Minimum sample size of 10 patients who underwent tooth extractions after radiotherapy in an H&N district
No previous ORN at the extraction site
Mean 6 months follow-up after tooth extractions
Unhealed sockets followed up for at least 3 months
**Exclusion criteria**
Case reports, reviews, cross-sectional studies
Studies in which no clear definition of ORN was reported
Studies not specifying whether ORN developed at the extraction site.

Studies on therapies of patients with ORN were included only if the ORN was effectively due to dental extractions and if the total number of patients receiving tooth extractions was clearly stated. Because many definitions of ORN have been proposed, confusion exists regarding its diagnosis, mainly concerning the time of bone exposure. The assessment of the period of bone exposure is crucial to achieving an ORN diagnosis, because it is not possible to clinically distinguish between a delayed alveolar bone healing and a true ORN. In this revision, studies without a clear definition of ORN were excluded to avoid biases. Abbreviations: ORN, osteoradionecrosis; RCTs, randomized clinical trials; H&N, head and neck.

## Data Availability

Data are available upon request to the authors.

## Supplementary Materials

The following are available online at https://www.mdpi.com/article/10.3390/cancers13225798/s1, File S1: Modified Newcastle-Ottawa quality assessment tool forms for observational studies, Table S1: Articles excluded from systematic review and reasons for their exclusions, Table S2: Details of reported ORN Patients. Gender, Age, and Tumor Site of ORN Patients are not reported because only a few data were retrieved.
