# Supplementary material for: Osteoradionecrosis of the Jaws Due to Teeth Extractions during and after Radiotherapy: A Systematic Review
Source: Cancers (Basel). 2021 Nov 18;13(22):5798. doi: 10.3390/cancers13225798 (PMC8616343; doi:10.3390/cancers13225798)
Supplement: Supplementary file 1 [file cancers-13-05798-s001.zip › cancers-1436075-supplementary.pdf]

ELECTRONIC MATERIALS

File S1. Modified Newcastle-Ottawa quality assessment tool forms for case control studies and cohort studies.

MODIFICATION OF NEWCASTLE NEWCASTLE - OTTAWA QUALITY ASSESSMENT SCALE - CASE CONTROL STUDIES

Note: A study can be awarded a maximum of one star for each numbered item within the Selection and Exposure categories. A maximum of two stars can be given for Comparability.

Selection

1) Is the case definition adequate?
   a) yes, with independent validation ⚜
   b) yes, e.g. record linkage or based on self reports
   c) no description
2) Representativeness of the cases
   a) consecutive or obviously representative series of cases ⚜
   b) potential for selection biases or not stated
3) Selection of Controls
   a) community controls ⚜
   b) hospital controls
   c) no description
4) Definition of Controls
   a) no history of disease (tooth extraction) ⚜
   b) no description of source

Comparability

1) Comparability of cases and controls on the basis of the design or analysis
   a) study controls for Tooth extraction ⚜
   b) study controls for any additional factor (Field of Radiation, Timing, Extraction Protocol, Reason for Tooth Extraction) ⚜

Exposure

1) Ascertainment of exposure
   a) secure record (e.g. surgical records) ⚜
   b) structured interview where blind to case/control status ⚜
   c) interview not blinded to case/control status
   d) written self report or medical record only
   e) no description
2) Same method of ascertainment for cases and controls
   a) yes ⚜

b) no

3) Non-Response rate

a) same rate for both groups ⚜

b) non respondents described

c) rate different and no designation

MODIFICATION OF NEWCASTLE NEWCASTLE - OTTAWA QUALITY ASSESSMENT SCALE - COHORT STUDIES

Note: A study can be awarded a maximum of one star for each numbered item within the Selection and Outcome categories. A maximum of two stars can be given for Comparability

Selection

1) Representativeness of the exposed cohort
   a) truly representative of the average of patients with history of head & neck cancer who undergo radiotherapy ⚜
   b) somewhat representative of the average of patients with history of head & neck cancer who undergo radiotherapy ⚜
   c) selected group of users e.g. nurses, volunteers
   d) no description of the derivation of the cohort
2) Selection of the non exposed cohort
   a) drawn from the same community as the exposed cohort ⚜
   b) drawn from a different source
   c) no description of the derivation of the non exposed cohort
3) Ascertainment of exposure
   a) secure record (e.g. surgical records) ⚜
   b) structured interview ⚜
   c) written self report
   d) no description
4) Demonstration that outcome of interest was not present at start of study
   a) yes ⚜
   b) no

Comparability

1) Comparability of cohorts on the basis of the design or analysis
   a) study controls for extraction ⚜
   b) study controls for any additional relevant factor (field of radiation, timing, extraction protocol, reason for tooth extraction) ⚜

Outcome

1) Assessment of outcome
   a) independent blind assessment ⚜
   b) record linkage ⚜
   c) self report
   d) no description
2) Was follow-up long enough for outcomes to occur
   a) yes (6 months from tooth extraction) ⚜
   b) no
3) Adequacy of follow up of cohorts
   a) complete follow up (at least 6 months) - all subjects accounted for ⚜
   b) subjects lost to follow up unlikely to introduce bias - small number lost - > 75 % (select an adequate %) follow up, or description provided of those lost) ⚜
   c) follow up rate < 75 (select an adequate %) and no description of those lost

d) no statement

Table S1. Articles excluded from systematic review and reasons for their exclusions.

**Article**	**Reason for exclusion**
Murray CG, 1980	No information about the number of patients undergoing tooth extractions
Murray CG, 1980	No/Incomplete definition of ORN is given
Murray CG, 1980	Case report
Beumer J, 1981	Review
Horiot JC, 1981	No/Incomplete definition of ORN is given
Coffin F. 1983.	No information about the number of patients undergoing tooth extractions
Horiot JC, 1983	No/Incomplete definition of ORN is given
Marciani RD, 1986.	Review
Makkonen TA, 1987	No/Incomplete definition of ORN is given
Epstein JB, 1987	Cohort of patients not representative of the whole patients undergoing tooth extractions prior to RT
Schweiger JW. 1987	Unhealed sockets followed up for less than 3 months
Kluth EV, 1988	Cohort of patients not representative of the whole patients undergoing tooth extractions prior to RT
Patel P, 1989	No/Incomplete definition of ORN is given
Levendag PC, 1989	No/Incomplete definition of ORN is given
Widmark G, 1989	Cohort of patients not representative of the whole patients undergoing tooth extractions prior to RT
Brown RS1, 1990	No/Incomplete definition of ORN is given
Reed JR. 1991	Less than 10 patients receive tooth extraction after RT
Katsikeris N, 1992	Letter to the editor

Ashamalla HL, 1996	Less than 10 patients receive tooth extraction after RT
Niewald M, 1996	No information about the number of patients undergoing tooth extractions
Roos DE, 1996	No clinical evaluation of the outcome ORN
Clayman L. 1997	Review
Curi MM, 1997	Cohort of patients not representative of the whole patients undergoing tooth extractions prior to RT
Epstein J, 1997	Cohort of patients not representative of the whole patients undergoing tooth extractions prior to RT
Carl W, 1998	Less than 10 patients receive tooth extraction after RT
Tong AC, 1999	Insufficient follow-up
Vudiniabola S, 1999	It is not specified if ORN followed post-RT tooth extractions. No data regarding patients who developed ORN
Thorn JJ, 2000.	Cohort of patients not representative of the whole patients undergoing tooth extractions prior to RT
Chavez JA, 2001.	No/Incomplete definition of ORN is given
Cramer CK, 2002	No clinical study
Sulaiman F, 2003	No/Incomplete definition of ORN is given
T. Reuther, 2003	Cohort of patients not representative of the whole patients undergoing tooth extractions prior to RT
Adkinson C, 2005	Cohort of patients not representative of the whole patients undergoing tooth extractions prior to RT
Bonan PR, 2006.	No/Incomplete definition of ORN is given
Cheng SJ, 2006	Cohort of patients not representative of the whole patients undergoing tooth extractions prior to RT

Studer G, 2006	No information about the number of patients undergoing tooth extractions
Chang DT, 2007	Less than 10 patients receive tooth extraction after RT
Goldwaser BR, 2007	No information about the number of patients undergoing tooth extractions
Lye KW, 2007	Insufficient follow-up
Chang DT, 2007	It is not specified if ORN followed post-RT tooth extractions
Harding SA, 2008	No clinical study
Jham BC, 2008.	Insufficient follow-up
Katsura K, 2008	No information about the number of patients undergoing tooth extractions
Koga DH, 2008	No/Incomplete definition of ORN is given
Sennhenn-Kirchner S, 2009	No information about the number of patients undergoing tooth extractions
Kaur J, 2009	No clinical study
Oh HK, 2009	Cohort of patients not representative of the whole patients undergoing tooth extractions prior to RT
Escoda-Francolí J, 2011	Less than 10 patients receive tooth extraction after RT
Gomez, D.R., 2011	No/Incomplete definition of ORN is given
Schuurhuis JM, 2011.	No information about the number of patients undergoing tooth extractions after RT
Chopra S, 2011.	Cohort of patients not representative of the whole patients undergoing tooth extractions prior to RT
Chiaojung Jillian Tsai, 2012	Less than 10 patients receive tooth extraction after RT
Thariat J, 2012	No clinical study

Duarte et al. 2013	It is not specified if ORN followed post-RT tooth extractions
Niewald M, 2013	Unclear number of post-RT tooth extraction
Heyboer M 3rd, 2013	No/Incomplete definition of ORN is given
Mozzati M, 2014	Insufficient follow-up
Walter C, 2014	It is not specified if ORN followed post-RT tooth extractions
Chronopoulos A, 2015	Cohort of patients not representative of the whole patients undergoing tooth extractions prior to RT
Raguse J-D, 2015	No/Incomplete definition of ORN is given
De Felice F, 2016	Cohort of patients not representative of the whole patients undergoing tooth extractions prior to RT
De Maesschalck et al. 2016	It is not specified if ORN followed post-RT tooth extractions
Kuhnt T, 2016	No information about the number of patients undergoing tooth extractions after RT
Kuo TJ, 2016	Only ORN with moderate and severe pain were diagnosed
Owosho 2016	Cohort of patients not representative of the whole patients undergoing tooth extractions prior to RT
Gallegos-Hernández JF, 2016	No information about the number of patients undergoing tooth extractions after RT
Wanifuchi S, 2016	Cohort of patients not representative of the whole patients undergoing tooth extractions prior to RT
Caparrotti F, 2017	No information about the number of patients undergoing tooth extractions after RT
Kojima Y, 2017.	Unclear follow-up
Lai TY, 2017	Tooth extractions were conducted even on patients who have not received RT. Impossible to evaluate the ORN rate.

Lalla RV, 2017	Ongoing Study
Moon DH, 2017	No information about the number of patients undergoing tooth extractions after RT
Beech NM, 2017	Cross-sectional study

Table S2. Details of reported ORN Patients. Gender, Age and Tumor Site of ORN Patients are not reported because only few data were retrieved.

Author	ORN Patients	Dose (Gy)[1]	Time RT/EXT	ORN Site	Preventive Measures
Morrish 1981[3]	1	73[2]	NA	Mandible	none
	2	73[2]	NA	Mandible	none
	3	73[2]	NA	Mandible	none
	4	73[2]	NA	Mandible	none
	5	73[2]	NA	Mandible	none
	6	73[2]	NA	Mandible	none
	7	73[2]	NA	Mandible	none
	8	73[2]	NA	Mandible	none
	9	73[2]	NA	NA[3]	none
Beumer 1983	10	NA	13-24 months	Maxilla	none
	11	NA	13-24 months	Maxilla	none
	12	NA	13-24 months	Maxilla	none
	13	NA	NA	Mandible (anterior)	none
	14	NA	NA	Mandible (anterior)	none
	15	NA	NA	Mandible (premolar)	none
	16	NA	NA	Mandible (premolar)	none
	17	NA	NA	Mandible (premolar)	none

	18	NA	NA	Mandible (premolar)	none
	19	NA	NA	Mandible (premolar)	none
	20	NA	NA	Mandible (premolar)	none
	21	NA	NA	Mandible (molar)	none
	22	NA	NA	Mandible (molar)	none
	23	NA	NA	Mandible (molar)	none
	24	NA	NA	Mandible (molar)	none
	25	NA	NA	Mandible (molar)	none
Marx 1985	26	68[2]	NA	Mandible	HBO
	27	68[2]	NA	Mandible	HBO
	28	68[2]	NA	Mandible	Penicillin V +Phenoxymethylpenicillin
	29	68[2]	NA	Mandible	Penicillin V +Phenoxymethylpenicillin
	30	68[2]	NA	Mandible	Penicillin V +Phenoxymethylpenicillin
	31	68[2]	NA	Mandible	Penicillin V +Phenoxymethylpenicillin
	32	68[2]	NA	Mandible	Penicillin V +Phenoxymethylpenicillin
	33	68[2]	NA	Mandible	Penicillin V +Phenoxymethylpenicillin
	34	68[2]	NA	Mandible	Penicillin V +Phenoxymethylpenicillin

	35	68[2]	NA	Mandible	Penicillin V +Phenoxymethylpenicillin
	36	68[2]	NA	Mandible	Penicillin V +Phenoxymethylpenicillin
	37	68[2]	NA	Mandible	Penicillin V +Phenoxymethylpenicillin
	38	68[2]	NA	Mandible	Penicillin V +Phenoxymethylpenicillin
Epstein 1987	39	53[2]	NA	Mandible	Antibiotic (NOS)
	40	53[2]	NA	Mandible	Antibiotic (NOS)
	41	53[2]	NA	Mandible	Antibiotic (NOS)

Footnote:

- [1] At Cancer site
- [2] Mean
- [3] Among the nine ORN, eight surely developed in the mandible.
- NA: Not Available
- NOS: Not Otherwise Specified
